# Whole Exome Sequencing Identifies New Host Genomic Susceptibility Factors in Empyema Caused by *Streptococcus pneumoniae* in Children: A Pilot Study

**DOI:** 10.3390/genes9050240

**Published:** 2018-05-03

**Authors:** Antonio Salas, Jacobo Pardo-Seco, Ruth Barral-Arca, Miriam Cebey-López, Alberto Gómez-Carballa, Irene Rivero-Calle, Sara Pischedda, María-José Currás-Tuala, Jorge Amigo, José Gómez-Rial, Federico Martinón-Torres, on behalf of GENDRES Network

**Affiliations:** 1 Unidade de Xenética, Departamento de Anatomía Patolóxica e Ciencias Forenses, Instituto de Ciencias Forenses, Facultade de Medicina, Universidade de Santiago de Compostela, 15782 Santiago de Compostela, Spain; j.pardoseco@gmail.com (J.P.-S.); barralarcaruth@gmail.com (R.B.-A.); Alberto.Gomez.Carballa@sergas.es (A.G.-C.); sara.pischedda91@hotmail.it (S.P.); mjcurras@gmail.com (M.-J.C.-T.); jorge.amigo@usc.es (J.A.); 2 GenPoB Research Group, Instituto de Investigaciones Sanitarias (IDIS), 15706 Santiago de Compostela, Spain; 3 Hospital Clínico Universitario de Santiago (SERGAS), 15706 Santiago de Compostela, Spain; 4 Translational Pediatrics and Infectious Diseases, Hospital Clínico Universitario de Santiago, 15706 Santiago de Compostela, Spain; Miriam.Cebey.Lopez@sergas.es (M.C.-L.); Irene.Rivero.Calle@sergas.es (I.R.-C.); Jose.Gomez.Rial@sergas.es (J.G.-R.); federicomartinon@gmail.com (F.M.-T.); 5 GENVIP Research Group (www.genvip.org), 15706 Santiago de Compostela, Spain

**Keywords:** *Streptococcus pneumoniae*, infectious disease, pediatrics, whole exome sequencing, next generation sequencing, parallel sequencing, transcriptome

## Abstract

Pneumonia is the leading cause of death amongst infectious diseases. *Streptococcus pneumoniae* is responsible for about 25% of pneumonia cases worldwide, and it is a major cause of childhood mortality. We carried out a whole exome sequencing (WES) study in eight patients with complicated cases of pneumococcal pneumonia (empyema). An initial assessment of statistical association of WES variation with pneumonia was carried out using data from the 1000 Genomes Project (1000G) for the Iberian Peninsula (IBS) as reference controls. Pseudo-replication statistical analyses were carried out using different European control groups. Association tests pointed to single nucleotide polymorphism (SNP) rs201967957 (gene *MEIS1*; chromosome 2; *p*-value_IBS_ = 3.71 × 10^−13^) and rs576099063 (gene *TSPAN15*; chromosome 10; *p*-value_IBS_ = 2.36 × 10^−8^) as the best candidate variants associated to pneumococcal pneumonia. A burden gene test of pathogenicity signaled four genes, namely, *OR9G9*, *MUC6*, *MUC3A* and *APOB*, which carry significantly increased pathogenic variation when compared to controls. By analyzing various transcriptomic data repositories, we found strong supportive evidence for the role of *MEIS1, TSPAN15* and *APOBR* (encoding the receptor of the *APOB* protein) in pneumonia in mouse and human models. Furthermore, the association of the olfactory receptor gene *OR9G9* has recently been related to some viral infectious diseases, while the role of mucin genes (*MUC6* and *MUC3A*), encoding mucin glycoproteins, are well-known factors related to chronic obstructive airway disease. WES emerges as a promising technique to disentangle the genetic basis of host genome susceptibility to infectious respiratory diseases.

## 1. Introduction

Pneumonia is the seventh reason of death in the USA and the leading cause of all infectious diseases [1,2]. In the pre-antibiotic era, *Streptococcus pneumoniae* was responsible for approximately 95% of all cases of pneumonia, and it remains responsible for 25% of all cases worldwide [3]. Complications of pneumococcal pneumonia used to be relatively uncommon; however, over the last years an increased incidence of complicated community-acquired pneumonia in children (mainly due to *S. pneumoniae*) has been referred [4]. This rise has been specifically linked to pneumococcal strains not included in the—by then only available—pneumococcal conjugate vaccine, and thus, to a replacement phenomenon [4,5,6]. This trend is being reversed by the new conjugate pneumococcal vaccines that include the causing serotypes [7]. The clinical features and course of complicated forms of pneumococcal pneumonia in children are quite characteristic and homogeneous; and despite the course of the disease is slow and cumbersome, it typically has a favorable outcome [8,9]. The majority of these cases occur mainly in otherwise previously healthy children without any identifiable risk factor [4,10]. The pneumococcal serotypes responsible for complicated forms usually involve serotypes 1, 3, 7F, 14 and 19A.

We here hypothesize that complicated forms of pneumococcal pneumonia due to specific serotypes in certain children might be related—among others—to host genetic factors. There are only a few studies that have aimed to analyze the genomic predisposition of the host to infectious diseases in children [11,12,13], and only recently, there begins to emerge suggestive evidence indicating host genetic factors involved in predisposition to pneumonia. Some of these studies focused in host genetic susceptibility to the invasive pneumococcal infection, indicating that mutations in genes involved in interleukin-1 receptor/Toll-like receptor (IL-1R/TLR) signaling pathway could be associated with this phenotype. More specifically, mutations in genes *IRAK4* and *MYD88* act disrupting IL-1R/TLR receptor signaling seem to be linked with pyogenic encapsulated bacterial infection in childhood, and more particularly, with invasive pneumococcal disease [14]. In a recent meta-analysis by Patarčić et al. [15], the authors found a single nucleotide polymorphism (SNP) located in gene *IL4* significantly associated to pooled respiratory infections, including pneumonia. By meta-analyzing genes related to host immune response in pneumonia development and progression, Smelaya et al. [16] reported the SNP rs5743708 (located in the proinflammatory cytokine gene *IL6*) as associated with severe sepsis/septic shock/severe systemic inflammatory response; while variation at rs18000896 (located in the anti-inflammatory cytokine gene *IL10*) was found to be a protector factor against the mentioned clinical phenotypes.

Several genomic strategies have been explored in the field of infectious diseases to investigate host genome susceptibility factors. Recently, whole exome sequencing (WES) has allowed to reveal new candidate SNPs and genes associated to respiratory syncytial virus (RSV) infections [13]. In contrast to other more popular genomic strategies strongly relying on linkage disequilibrium to capture candidate risk variation (e.g., genome wide association studies or GWAS; [11,12,17]), WES allows to discover new genome variation related to disease by focusing on protein-altering variants, which are supposed to be enriched for causal effects [18]. Then, variation observed in exomes of patients can be compared to variation observed in healthy controls. The focus on complicated forms of the disease contributes to increase the statistical power of the study under the assumption that these individuals most likely carry genetic variants that have higher effect, or their exomes are enriched with more pathogenic variation than expected in moderated or mild phenotypes. The burden of the pathogenic variation accumulated in the genomes of patients can be examined by using new statistical procedures employing algorithms that take into account theoretical predictions of pathogenicity [19,20,21].

We aimed at revealing host genetic factors involved in complicated forms of pneumonia, concretely with empyema caused by *S. pneumoniae*, using WES. The best candidate genes from WES data were further investigated in transcriptomic repositories to search for further evidence of their association with empyema.

## 2. Materials and Methods

### 2.1. Study Design and Inclusion Criteria

Patients were selected from the GENDRES network (Genetic, vitamin D and respiratory infections research network [22], which is a cohort prospectively recruited through an observational study run in Spain through a national hospital-based research network for pediatric respiratory research. The GENDRES network includes 13 Spanish tertiary hospitals (see also [13,23,24]). Selected patients from the GENDRES cohort have been used recently in other clinical and genomic studies on other pathogen-caused diseases [13]. 

Any patient from 1 month to 14 years of age admitted to any of the GENDRES network hospitals with confirmed pneumococcal empyema was eligible, provided that (i) written informed consent was available, (ii) at least a nasopharyngeal and DNA sample was collected, and (iii) the minimum mandatory demographic and clinical data set was recorded. 

Pneumonia was defined as an inflammation of one or both lungs lobar or segmental or multi-lobar collapse/consolidation on chest X ray with clinical compatible symptoms [25,26]. Empyema was defined as the presence of grossly purulent fluid in the pleural cavity; in practice: (i) thoracentesis with microbial growth from pleural fluid; or (ii) thoracentesis with no growth on culture of pleural fluid but elevated protein, or cell count (normal and abnormal reference values as determined by the clinical laboratory at each center); (iii) ultrasound or other diagnostic imaging evidence of pleural fluid assessed by the radiologist as empyema, or (iv) diagnosis at time of thoracic surgery. Pneumococcal etiology was established through culture and/or polymerase chain reaction (PCR) identification in sterile specimens (i.e., blood or pleural fluid) [25,26].

For the present sub-study, eight children with confirmed diagnosis of pneumococcal pleural effusion were finally selected. A blood sample was collected from patients as early as possible during the patient’s assessment. DNA for WES analysis was extracted using Wizard Genomic DNA Purification Kit (Promega, Fitchburg, Wisconsin, United States) and following the recommended protocol.

The study was approved by the Ethical Committee of Clinical Investigation of Galicia (CEIC ref 2010/015) and by the regional ethics committees for each participating Spanish center. Written informed consent was obtained from a parent or legal guardian for each subject before study inclusion.

Genomic variation obtained from cases was contrasted against healthy controls. Ancestry European matched controls were collected from different genome reservoirs for genomic and statistic comparisons (see details below on data-mining).

### 2.2. Whole Exome Sequencing

Enrichment and library preparation of samples were carried out as done before [13]. Briefly, samples were initially prepared following the Agilent’s SureSelect Protocol v.1.2 (Agilent, Santa Clara, CA, USA), and enrichment according to Agilent SureSelect protocols. The Agilent’s QPCR NGS Library Quantification Kit (G4880A) was used to measure concentration of the libraries. Samples were pooled prior to sequencing with each sample at a final concentration of 10 nM. Sequencing was performed on the Illumina HiSeq2000 platform (Illumina, San Diego, CA, USA) using TruSeq v3 chemistry (Illumina, San Diego, CA, USA). Read files (Fastq) were generated from the sequencing platform using manufacturer’s proprietary software. Reads were mapped to the human genome hg19/b37 using the Burrows-Wheeler Aligner (BWA) package v.0.6.2 [27]. Local realignment of the mapped reads around potential insertion/deletion (indel) sites was carried out with the Genome Analysis Tool Kit (GATK) v.1.6 [28]. Duplicate reads were marked using Picard v.1.104. Samtools v.0.1.18 [29] was also used to work with BAM files and base quality (Phred scale) scores were recalibrated using GATK’s covariance. The average read length was 100 bp.

A minimum of 86% of the on-target regions were covered to a depth of at least 20 times. Exome sequencing was carried out in Oxford Gene Technology [30]. The raw data was entirely processed in the laboratory in Santiago de Compostela, Spain.

As a quality control of massive parallel sequence results we processed the same sample twice following the same steps as described in [13]. The two exome sequences were compared, and the coincidence of the sequencing results was 99.999%. 

### 2.3. Annotation of Variants and Assessment of Their Pathogenicity

We followed the same methodological procedures described previously [13]. Briefly, GATK v3.4 [28] was used for variant detection for multi-sample calling. The HaplotypeCaller algorithm [28] was used to obtain the genomic VCF files algorithm, and the GenotypeGVCFs [28] algorithm to carry out joint genotyping. VQSR algorithm [28] was used to recalculate variant quality scores. Variants were annotated using ANNOVAR [31], and using gene and gene function data from Ensembl [32]

There exist different scoring systems for annotated variants that measure the pathogenicity/deleteriousness of SNPs, such as PolyPhen [19], SIFT [20], or GERP [21]. We used Combined Annotation Dependent Depletion (CADD) [33]. Compared to other scores, CADD integrates multiple annotations by contrasting variants that survived natural selection with simulated mutations [33]. This score quantitatively prioritizes functional, deleterious, and disease-causal variants across a wide range of functional categories, effect sizes and genetic architectures. The linear kernel support vector machine-based algorithm used in CADD analysis has been improved by using a deep neural network, which also considers nonlinear effects. This modified CADD algorithm is now known as deleterious annotation of genetic variants (DANN); it also provides a score [34].

### 2.4. Statistical Analysis of Whole Exon Sequencing Data

Several analyses were initially performed to investigate the population characteristics of patients in regard to their ancestry. The aim of these analyses is to detect possible genome outliers that could increase the false positive rate in association tests. PLINK software [35] was first used to compute IBS values from SNP data. A multidimensional scaling (MDS) analysis was built from a matrix of pairwise individual IBS values computed on patients and individuals from reference continental populations. MDS analysis was carried out using R [36] and its library *stats* (function *cmdscale*) [36]. In addition, admixture patterns were investigated in our patients by contrasting their variation against genome data from populations representing main continental regions. 

We used data from the 1000 Genomes Project [37]; hereafter 1000G) as the main resource for reference European population and control individuals for association tests. Management of 1000G data was carried out using previous bioinformatics developments our group [38,39]. Potential familial relationships were also explored as done previously [40].

Two different association analyses were carried out. First, a Fisher’s exact test was computed on common variants, defining common as MAF > 5% in the 1000G Iberian sample set (1000G-IBS). These single-point association analyses were carried out considering the presence of population stratification using the inflation factor lambda (λ). Second, a burden test of pathogenicity by gene was undertaken by collapsing variants and using DANN score as covariant. In particular, we used the Weighted-Sum collapsing method by genes [41]. 

As controls, we used the 1000G-IBS for the discovery phase, and we performed pseudo-replication association tests using additional 1000G control groups of European ancestry [42,43], namely, Tuscany (1000G-TSI), Great Britain (1000G-GBR) and Europeans from Utah (1000G-CEU). Association tests were also carried out against all the 1000G controls merged into a single group (labelled as 1000G-ALL) in order to increase statistical power. In addition to the 1000G European controls, we also compared our cases with European controls from the Spanish exome control data (*n* = 267) of Dopazo et al. [44]. 

A conservative correction for multiple hypothesis tests was carried out using the Bonferroni adjustment for all association tests between cases and control groups, in both single-point association tests and burden tests of pathogenicity. Most of the computations were performed using in-house R and Perl scripts [45].

We used Haploview [46] to display linkage disequilibrium (LD) patterns between SNPs; in particular we used the D’ CI methods of Gabriel et al. [47] implemented in this software.

### 2.5. Statistical Analysis from Transcriptome Data

To identify transcript signatures in patients with differing pneumococcal diseases we interrogated the GEO repository [48] for the queries: ‘Streptococcus pneumoniae’ OR/AND ‘pneumococcus’. We filtered the results from these queries by selecting only those studies on humans and mice. We detected only six studies containing suitable data to validate our gene candidates, including three studies in mice (GEO accession numbers: GSE42464 [49], GSE49533 [50], and GSE45644 [51], all of them carried out in lung tissue), and three in humans (GSE49755 [52] in plasma, GSE6269 [53], in blood, and GSE58291 [54], in corneal tissue). Table S1 contains the microarray sample IDs of the datasets analyzed that passed all the quality filters and were publicly available (February 10, 2018). We did not find signals of differential expression of our candidate genes in the study by Ramilo et al. [53] (GSE6269), therefore this study did not receive further attention in our analyses. A brief summary of the five studies of interest is provided in Supplementary Text.

First the quality of the raw microarray data compiled from the five mentioned studies were analyzed using the R packages *lumi* [55] and *oligo* [56]. Next, to evaluate if our genes of interest were differentially expressed between patients with pneumococcal disease and controls, a linear model was fit, and moderated *t*-statistics was calculated for each transcript. Correction for multiple test was carried out using the false discovery rate method by Benjamini and Hochberg’s and employing the R package *limma* [57].

We found signals of altered expression in three genes of interest, *MEIS1*, *TSPAN15*, and *APOBR*. Their performance as potential biomarkers was evaluated using receiver operating characteristic (ROC) curves that represent the true positive rate (TPR) against the false positive rate (FPR) at different threshold cut-off points.

## 3. Results

### 3.1. Clinical Characteristics of Patients

Eight children with confirmed diagnosis of pneumococcal empyema were finally selected among the GENDRES cohort. The patients’ main characteristics are summarized in Table 1. The mean (SD) age of the subjects studied was 5.1 (3.1) years. All the patients had been vaccinated with at least one pneumococcal vaccine and two of them had asthma as co-morbidity. Prior to admission, five patients had been treated with antibiotics or antipyretics. Children were hospitalized a mean (SD) of 21.2 (16.4) days and were 8.0 (3.8) days at pediatric intensive care unit (PICU). Two of the patients were transferred twice to the PICU during the current episode due to worsening of the illness. In all included patients, pneumococcal etiology was confirmed by blood and/or pleural culture or PCR.

### 3.2. Population Genetic Characteristics of Empyema Patients

Analysis of identity by descent (IBD) patterns did not reveal the existence of close relationships among patients. In order to detect possible population outliers that could interfere with association tests, we undertook several population-based analyses by comparing our cases with reference populations representing the main continental groups. 

**Figure 1:**
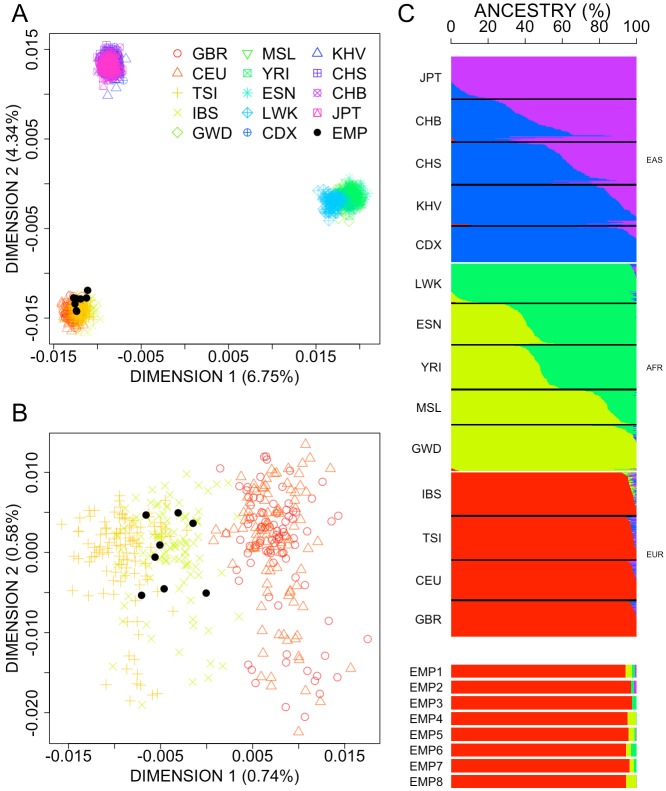
(**A**) MDS plot of pair-wise individual identity by state (IBS) values between cases vs. reference continental populations from 1000G. (**B**) MDS plot of cases and European 1000G reference populations [37]. (**C**) Analysis of admixture for the samples analyzed in (**A**). GBR: British in England and Scotland; CEU: Utah Residents (CEPH) with Northern and Western European Ancestry; TSI: Tuscany in Italia; IBS: Iberian Population in Spain; GWD: Gambian in Western Divisions in the Gambia; MSL: Mende in Sierra Leone; YRI: Yoruba in Ibadan, Nigeria; ESN: Esan in Nigeria; LWK: Luhya in Webuye, Kenya; CDX: Chinese Dai in Xishuangbanna, China; KHV: Kinh in Ho Chi Minh City, Vietnam; CHS: Southern Han Chinese; CHB: Han Chinese in Bejing, China; JPT: Japanese in Tokyo, Japan; EMP: pneumococcal empyema cases.

We first performed a MDS to a continental context using 1000G reference populations representing sub-Saharan Africa, East Asia, and Europe (Figure 1A). As expected, all the populations display along the vertexes of a triangle, and our cohort of patients clearly fit with the European pole of this plot in the first and second dimensions. To confirm this scenario, a second MDS analysis was carried out for the European samples alone (Figure 1B); this plot confirms the genomic proximity of the pneumonia patients with the 1000G-IBS dataset.

The results of an admixture analysis [58] corroborate the results obtained from the MDS analysis (Figure 1C), indicating that all the pneumonia patients have virtually 100% European ancestry. 

Overall, we found no evidence of population stratification in both cases and controls.

### 3.3. Single Nucleotide Polymorphism Association Test

**Figure 2:**
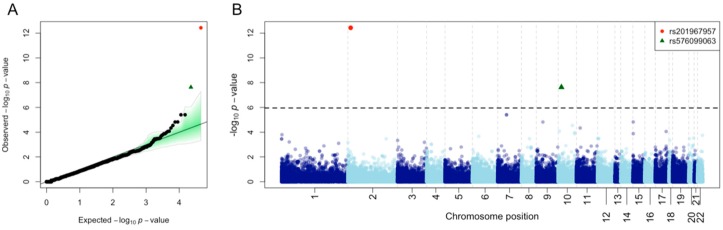
(**A**) Quantile-quantile (QQ)-plot of *p*-values for common variation observed in patients against 1000G-IBS controls. The green shadow indicates the *p*-values obtained under a permutation approach (1000 permutations). (**B**) Manhattan plot of common variants observed in patients against 1000G-IBS controls. The dotted line indicates the Bonferroni threshold.

**Figure 3:**
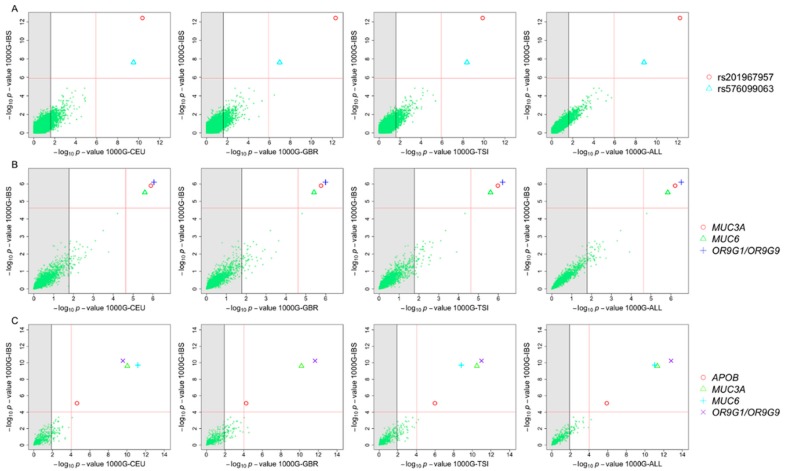
(**A**) *p*-values of association tests carried out between cases and different 1000G control groups computed on single nucleotide polymorphisms (SNPs). (**B**) *p*-values of gene burden association tests between patients and controls using common variants. (**C**) *p*-values of gene burden association tests between patients and controls using rare variants (minor allele frequency, MAF < 0.05 for the 1000G-IBS cohort). The grey shadow to the left of each individual graph indicates the threshold for the corresponding Bonferroni adjustments according to the number of candidate SNPs/genes. The red lines indicate the genomic Bonferroni threshold for the two control groups being compared in each graph. The legend on the right indicates the SNPs/genes surpassing the genomic Bonferroni’s thresholds.

Annotation of WES data yielded 118,690 sequence variants. A description of the functional characteristics of these variants is provided in Table 2. Single-point association tests were carried out for common variants (*n* = 44,941). A quantile-quantile (QQ)-plot of the *p*-values for this common variation indicates a good fit with expectations according to a uniform distribution (built on 1000 permutations), with the exception of two variants located at the top tail end of the plot, which yielded *p*-values below the expected values (Figure 2A). A Manhattan plot of all the WES common variation allows to visualize that these two SNPs have *p*-values well below the Bonferroni threshold when using 1000G-IBS as controls (Figure 2B). Additional association tests using other 1000G European control sets surpassed the Bonferroni statistical significance threshold (Table 3; Figure 3). The inflation factor was below 1 for these analyses, so correction for population stratification was not needed.

The SNP rs201967957 (G/A) located at gene *MEIS1* was the most significant when compared against 1000G-IBS (*p*-value_IBS_ = 3.71 × 10^−13^; OR = 145.5). The second most significant SNP was rs576099063 (*p*-value_IBS_ = 2.36 × 10^−8^; OR = 26.6), located at gene *TSPAN15*. The two SNPs are located in the untranslated regions of the mentioned genes (5′-UTR and 3′-UTR, respectively). These gene regions are generally related to the regulation of the expression in eukaryotic organisms. 

We also carried out association tests by collapsing all variants in genes and taking into account their accumulated pathogenicity. Three genes showed statistical significance when compared to 1000G-IBS (Table 4), namely, *OR9G9* (*p*-value_IBS_ = 8.13 × 10^−7^), *MUC3A* (*p*-value_IBS_ = 1.27 × 10^−6^) and *MUC6* (*p*-value_IBS_ = 3.16 × 10^−6^). Subsequently, we repeated the burden association test but focusing on rare variants exclusively. Gene *APOB* (*p*-value_IBS_ = 8.35 × 10^−6^) appeared as statistically associated on top of the other three genes (Table 4). 

The burden test analyses were pseudo-replicated using the other European control groups from 1000G; and in all comparisons, the same genes appeared as statistically significant (Table 4). Furthermore, the same genes showed statistically significant values for the Spanish control group in Dopazo et al. [44]. 

Patterns of linkage disequilibrium (LD) for the candidate genes inferred from single SNP association test and burden test are displayed in Figures S1 and S2.

### 3.4. Transcription Signatures of Main Candidate Genes

Analysis of exome data raised six good candidate genes statistically associated with pneumonia. Data from large transcriptomic repositories were investigated for the six genes for which signals of statistical genomic association were observed between pneumonia patients and controls, namely, two genes in single-point association tests and four genes in burden tests.

**Figure 4:**
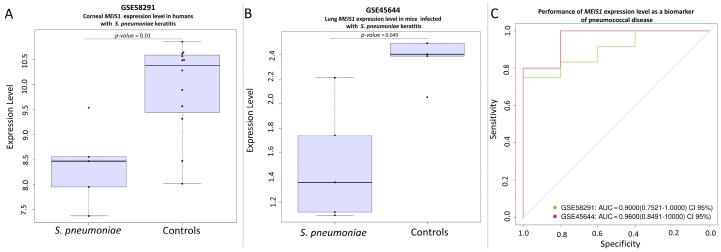
(**A**) Differential expression level of the *MEIS1* gene between corneal tissue from corpses and corneal tissue from *S. pneumoniae* keratitis patients in the study GSE58291. (**B**) Differential lung expression level of *MEIS1* gene between healthy mice and *S. pneumoniae* infected mice in the study GSE45644. (**C**) Receiver operating curve (ROC evaluating the potential of the gene *MEIS1* as a biomarker in the studies GSE58291, and GSE45644. AUC: area under the curve.

**Figure 5:**
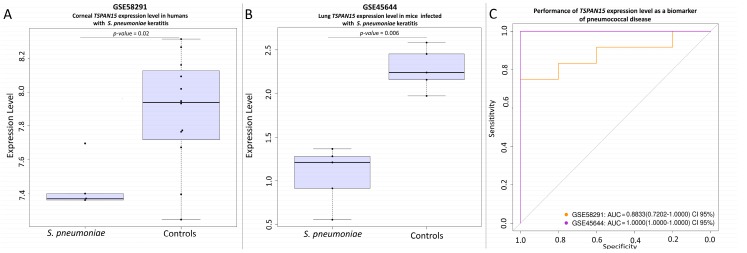
(**A**) Differential expression level of the *TSPAN15 gene* between corneal tissue from corpses and corneal tissue from *S. pneumoniae* keratitis patients in the study GSE58291. (**B**) Differential lung expression level of *TSPAN15* gene between healthy mice and *S. pneumoniae* infected mice in the study GSE45644. (**C**) ROC evaluating the potential of the gene *TSPAN15* as a biomarker in the studies GSE58291, and GSE45644.

The gene *MEIS1* was found to be down-regulated in both corneal tissue from humans (GSE58291 [54]; Figure 4A) and lung tissue from mice (GSE45644 [51]; Figure 4B) suffering pneumonia caused by *S. pneumoniae*. In mice, the *p*-value is in the multiple-test correction limit, which could reflect the low sample size available for cases and controls. *TSPAN15* shows a similar behavior as *MEIS1*. It seems to be down-regulated in humans (Figure 5A) and in mice (Figure 5B) when compared to their respective controls in the same studies (GSE58291 and GSE45644; respectively). 

**Figure 6:**
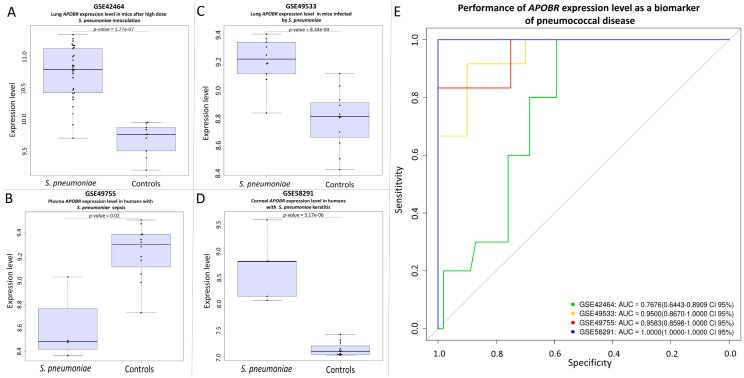
(**A**) Differential lung expression level of the *APOBR* gene between healthy mice and *S. pneumoniae* infected mice in the study GSE42464. (**B**) Plasma expression level of the *APOBR* gene between healthy human control and *S. pneumoniae* sepsis patients in the study GSE49755. (**C**) Lung expression level of the *APOBR* gene between healthy mice and *S. pneumoniae* infected mice in the study GSE49533. (**D**) Differential expression level of the *APOBR* gene between corneal tissue from corpses and *S. pneumoniae* keratitis patients in the study GSE58291. (**E**) ROC evaluating the potential of the *APOBR* gene as a biomarker in the studies GSE42464, GSE49755, GSE49533, and GSE58291.

We found a significant result of association for the transcript of *APOBR* gene in four studies (GSE42464 [49], GSE49533 [50], GSE49755 [52], and GSE58291 [54]). The *APOBR* encodes the receptor of the *APOB* protein. The transcription signal observed is strong in both mice and humans (Figure 6). Interestingly, we found that *APOBR* appears as up-regulated when investigating cornea of infected patients and infected mice lung tissues, but down-regulated in plasma.

The performance of the genes as potential biomarkers was evaluated by generating ROC curves (Figure 4 to Figure 6). The area under the curve (AUC) was greater than 75% in all the studies examined.

## 4. Discussion

Pediatric pneumococcal pneumonia mostly occurs in otherwise healthy children [10,59]. Several factors have previously been pointed out to explain this clinical phenotype but not genetic predisposition [4,10,59,60]. According to the results of our WES-based approach in the setting of pneumococcal empyema in previously healthy children, host genetic factors might contribute to explain the complex pathophysiology of this important clinical phenotype. 

Antibiotic resistance does not seem to be a key factor in the pathogenesis of complicated pneumococcal pneumonia. Curiously, pneumococcal empyema is less likely to be caused by penicillin-resistant pneumococci than uncomplicated pneumococcal pneumonia, showing that antibiotic sensitivity or resistance is not a major factor in the course of complicated pneumococcal pneumonia [4,6,61,62,63].

Results from WES indicate that there are two SNPs statistically associated in empyema patients. The two SNPs fall in strong blocks of LD (Figure S1), meaning that other SNPs located in the same genes (*MEIS1* and *TSPAN15*) could be responsible for the association observed. In addition, WES data also revealed four candidate genes with unexpected amounts of accumulated pathogenicity in pneumonia patients. The six genes are particularly interesting because they code for proteins that have been previously linked to infectious diseases. 

The nasopharynx, one of the natural entrances of *S. pneumoniae* to the host, is particularly rich in mucin proteins. Therefore, detection of *MUC6* (mucin 6, oligomeric mucus/gel-forming) and *MUC3A* (mucin 3A, cell surface associated) as associated to pneumonia seems most relevant. Mucin encodes epithelial glycoproteins, and its expression related to airway diseases has been reported in the literature [64]. Mucin glycoproteins are secreted in large quantities by mucosal epithelia and they play important roles by limiting infectious gastrointestinal and respiratory diseases [65]. Yesilkaya et al. [64] showed results pointing to mucins as key factor in the virulence gene expression in *S. pneumoniae*. Furthermore, the association of *MUC6* gene with pneumonia observed in our patients has recently been associated with RSV disease [13]. RSV and pneumococci may reciprocally and synergistically collaborate when infecting the host, contributing to disease severity [66,67]. Interestingly, this shared feature on gene *MUC6* might also point to a common host genetic predisposition to both infections.

Some variants of *TSPAN15* gene have been associated with lung damage [68]. The gene family of tetraspanins are involved in a variety of molecular processes including migration, adhesion, signaling and pathogen infections [69,70]. Tetraspanin CD9 negatively regulates lipopolisaccaride response in terms of macrophage activation and lung inflammation in mice models and statins might exert anti-inflammatory effects by unregulating tretraspanin CD9 [71,72].

The connection of apolipoprotein B (encoded by gene *APOB*) with respiratory disease has already been suggested in the literature. In addition, Peterson et al. [73] found that homeostatic levels in blood of the APOB protein represents an innate barrier against invasive *Staphylococcus aureus* infection. 

The large repository of expression data GEO was explored for data related to pneumonia caused by *S. pneumoniae*. We were able to retrieve expression data of interest that focused in mice and humans. However, the fact that only five small studies were available denotes that the field is still very incipient. Interestingly, and despite the low sample sizes of the targeted studies, all of them indicate that *S. pneumoniae* modifies the transcriptome of the host. We were able to detect altered patterns of host expression for three out of our six candidate genes. Thus, *MEIS1* and *TSPAN15* genes were found to be down-regulated in humans and mice as a result of *S. pneumoniae* infections. With regard to gene *APOB*, we did not find an overrepresentation of its transcript in these transcriptomic studies, but we found overexpression signals of the transcript generated by the gene encoding the apolipoprotein B receptor (*APOBR*). The direct molecular link existing between *APOB* and *APOBR* is very suggestive of an association of pneumonia caused by *S. pneumoniae* and the APOB pathway. Furthermore, we have observed a tissue-dependent regulation in the data from different studies, three of them showing over-expression of *APOBR* in lung [49,50] and cornea [54], (two tissues that are directly exposed to the air and therefore a possible direct contact with the pathogen), while another study indicates a down-regulation of *APOBR* in plasma [52]. This result seems most promising for the understanding of the mechanisms of infection of *S. pneumoniae*, but it needs further validation in larger cohorts.

Last but not least, the transcription signals we identified for *MEIS1*, *TSPAN15*, and *APOBR* genes have been found in both mice and humans. This suggests a highly conservative evolutionary mechanism of infection in *S. pneumoniae*. In addition, the data suggest that the role of these genes is not tissue-dependent, since we have observed differential expression signals in plasma, lung, and corneal tissues. ROC curves also show that these genes can be reliable in clinical diagnostic applications; this however requires further validation and their potential utility would most likely result from their combination with more biomarkers.

There are several limitations in the present study. On the one hand, the cohort of patients analyzed is limited and therefore, the statistical genomic findings need further validation in independent cohorts. We have tried to overcome this limitation by analyzing extreme phenotypes of pneumonia and using several control groups for pseudo-replication, for which the results were consistent. Another limitation comes from the expression data available in the literature, which is also very limited in terms of number of studies and sample sizes. Moreover, some studies use different platforms or expression arrays. For instance, in the study [53], controls were analyzed with the Affymetrix Human Genome U133A Array, whereas the *S. pneumoniae* cases were analyzed with three different arrays (Affymetrix Human Genome U133A Array, Affymetrix Human Genome U133 Plus 2.0 Array, and the Sentrix Human-6 Expression BeadChip). The use of different platforms or arrays might limit our ability to explore the transcription signals of interest. Moreover, in the same study, the patients analyzed were treated with antibiotics before sample collection, which could most likely alter the transcriptomic of a patient infected by *S. pneumoniae*.

It is however relevant that, despite the small sample sizes and the other technical limitations, we observed supportive results for three out of the six WES candidate genes.

To the best of our knowledge this is the first study that uses next generation sequencing (NGS) techniques and WES in the context of pneumonia, and one of the very few in the wider area of infectious disease in childhood. By way of targeting severe phenotypes we were able to identify good gene candidates related to complicated forms of pneumococcal pneumonia. Furthermore, our findings provide new candidate biomarkers to be tested and validated in clinical settings.

## Figures and Tables

**Table 1:** Summary of demographic and clinical characteristics of the study cohort.

Variables	Pneumococcal Empyema Patients
**Demographic characteristics**	
Sex (male) ^a^	5/8 (62.5%)
Age (years) ^b^	5.1 (3.1)
**Medical history**	
Asthma ^a^	2/8 (25.0%)
**Pneumococcal vaccination status ^a^**	
**PCV**	6/8 (75.0%)
PCV 10	1/8 (12.5%)
PCV 13	1/8 (12.5%)
**Clinical data**	
**Treatment prior to admission ^a^**	
Antibiotics	3/8 (37.5%)
Antipyretic	2/8 (25.0%)
Hospital length of stay (days) ^b^	21.2 (16.4)
PICU (days) ^b^	8.0 (3.8)
Respiratory support ^a^	2/8 (25.0%)
Oxygen ^a^	4/8 (50.0%)
Urokinase ^a^	5/8 (62.5%)
**Blood test**	
Leukocytes (c/mm^3^) ^b^	17,971.2 (5751.5)
Procalcitonin (ng/mL) ^b^	253.5 (565.4)
**Pleural fluid test**	
Glucose (mg/dL) ^b^	19.4 (25.2)
Proteins (g/dL) ^b^	5.0 (1.0)
**Course and outcome**	
**Course ^a^**	
Necrotizing pneumonia	4/8 (50.0%)
Sepsis	1/8 (12.5%)
**Sequelae** ^a^	
Pneumatocele	1/8 (12.5%)
Exitus	1/8 (12.5%)

^a^ number of patients (%). ^b^ mean (standard deviation, SD); PICU: paediatric intensive care unit; PCV: Pneumococcal conjugate vaccine.

**Table 2:** Description of sequence variants found in the exomes of our patients.

Sequence Variation	*n*
Downstream	6
Exonic	76,551
Exonic/splicing	50
Intergenic	37
Intronic	349
ncRNA_exonic	5728
ncRNA_exonic; splicing	5
ncRNA_intronic	483
ncRNA_splicing	3
Splicing	48
Upstream	17
Upstream; Downstream	2
3′-UTR	10,687
5′-UTR	7124
5′-UTR5/3′-UTR	15
Non-synonymous SNV	38,911
Stopgain	368
Stoploss	40
Synonymous SNV	36,270
Unknown	1012

ncRNA: noncoding RNA; UTR: untranslated region; SNV: single nucleotide variant.

**Table 3:** Association test of the best two single nucleotide polymorphism (SNP) candidates: rs201967957 (G/A) located in gene *MEIS1* (chromosome 2) and rs576099063 (G/T) located in gene *TSPAN15* (chromosome 10). The table shows results for comparisons of cases versus different control groups, namely, 1000G-IBS, 1000G-GBR, 1000G-TSI, all these 1000G controls merged in a single group (abbreviated as ‘1000G-ALL’).

Cohort	Statistical Values	rs201967957 (G/A)	rs576099063 (G/T)
Cases	AF	0.9375	0.8125
1000G-IBS	MAF	0.09346	0.1402
	OR	145.50	26.58
	*p*-value	3.71 × 10^−13^	2.36 × 10^−8^
1000G-CEU	MAF	0.13920	0.08763
	OR	92.78	45.12
	*p*-value	4.40 × 10^−11^	3.07 × 10^−1^
1000G-GBR	MAF	0.08989	0.15730
	OR	151.90	23.21
	*p*-value	4.84 × 10^−13^	1.05 × 10^−7^
1000G-TSI	MAF	0.1557	0.1179
	OR	81.36	32.41
	*p*-value	1.34 × 10^−1^	4.22 × 10^−9^
1000G-ALL	MAF	0.12	0.1262
	OR	109.90	29.99
	*p*-value	6.05 × 10^−13^	1.60 × 10^−9^

AF: allele frequency in cases that is minor in controls; MAF: minor allele frequency; OR: odds ratio.

**Table 4:** Burden test of SNP considering all variants in genes and only those with low frequencies (MAF < 0.05 for the 1000G-IBS cohort). Average DANN per genes was used as covariant for the association test. The genes showing the lowest *p*-value against the 1000G-IBS control group were further tested in other control groups.

Genes	Chr.	No. SNP	*p*-Value_IBS_	*p*-Value_CEU_	*p*-Value_GBR_	*p*-Value_TSI_	*p*-Value_ALL_	*p*-Value_EC_
**All variants**								
*OR9G9*	11	17	8.13 × 10^−7^	8.94 × 10^−7^	1.02 × 10^−6^	6.04 × 10^−7^	3.05 × 10^−7^	*−*
*MUC3A*	7	45	1.27 × 10^−6^	1.27 × 10^−6^	1.70 × 10^−6^	1.05 × 10^−6^	6.15 × 10^−7^	8.94 × 10^−6^
*MUC6*	11	34	3.16 × 10^−6^	2.56 × 10^−6^	3.83 × 10^−6^	2.47 × 10^−6^	1.45 × 10^−6^	1.92 × 10^−6^
**Rare variants**								
*OR9G9*	11	11	5.62 × 10^−11^	2.74 × 10^−10^	2.21 × 10^−12^	1.04 × 10^−11^	1.58 × 10^−13^	*−*
*MUC6*	11	24	1.90 × 10^−10^	6.74 × 10^−12^	0	1.52 × 10^−9^	9.17 × 10^−12^	1.22 × 10^−8^
*MUC3A*	7	21	2.42 × 10^−10^	9.23 × 10^−11^	6.48 × 10^−11^	3.28 × 10^−11^	4.65 × 10^−12^	4.69 × 10^−9^
*APOB*	2	36	8.35 × 10^−6^	2.37 × 10^−5^	5.56 × 10^−5^	1.03 × 10^−6^	1.28 × 10^−6^	1.07 × 10^−6^

EC: data from the exome sequencing data of the Spanish control group (*n* = 267) in Dopazo et al. [44]; Chr: chromosome.

## Supplementary Materials

The following are available online at http://www.mdpi.com/2073-4425/9/5/240/s1, Table S1: ID and details of samples used for transcription analyses. Figure S1: LD patterns in gene *MEIS1* and *TSPAN15* for variants with MAF > 5%. The blue rectangles point to the best SNP candidates rs201967957 and rs576099063. Figure S2: LD patterns for all the SNPs observed in genes *MUC3A*, *MUC6*, *OR9G1*, and *APOB*.
