# Supplementary material for: Whole Exome Sequencing Identifies New Host Genomic Susceptibility Factors in Empyema Caused by Streptococcus pneumoniae in Children: A Pilot Study
Source: Genes (Basel). 2018 May 3;9(5):240. doi: 10.3390/genes9050240 (PMC5977180; doi:10.3390/genes9050240)
Supplement: Supplementary file 1 [file genes-09-00240-s001.zip › supplementary-resubmission.pdf]

Whole Exome Sequencing identifies new host genomic susceptibility factors in empyema caused by Streptococcus pneumoniae in children

Supplementary Text

The study by van Lieshout et al. [1] (GSE42464) investigated the role of two genes (*NLRP* and *ASC*) in mice suffering *S. pneumoniae* pneumonia, and discovered several genes differentially expressed between two mice strains, one deficient in *NLRP* and other for *ASC*. By performing a lung whole-genome transcriptional profiling with microarrays these authors observed different susceptibilities between mice strains after infection which was reflected by an increased bacterial dissemination and lethality in the ASC deficient mice strain.

Jonczyk et al. [2] (GSE49533) conducted a GWAS to map genetic loci associated with susceptibility to pneumococcal infection in different mice strains. Using microarrays and RT-PCR, these authors also analyzed the lung transcriptome between resistant and susceptible mouse strains during pneumococcal infection. The pulmonary transcriptome differed between both groups; an enrichment of genes involved in vascular remodeling and response to lesions was observed in both mice groups during the infection process.

In another study carried out in mice, Restori et al. [3] (GSE45644) analyzed pneumonia disease progression, acute-phase response, and lung gene expression fingerprints in mice inoculated with *S. pneumoniae*. Their microarray analysis of lung tissue showed significant changes in the acute-phase protein serum amyloid A levels between uninfected and infected mice; they also observed that these changes were reduced by immunization. In addition, they also noted that the immunization with PPS3 and also the co-immunization with PPS3 and LPS outstandingly reduced pneumonia severity.

Khaenam et al. [4] (GSE49755) analyzed plasma from patients with bacterial sepsis and from uninfected healthy controls to stimulate three blood cell types (neutrophils, mononuclear cells, and monocyte-derived dendritic cells). Microarrays were used to study the transcriptome of the stimulated cells. Neutrophils yielded the clearest result with a number of genes showing differential expression levels; many of those genes were involved in well-known

immunologic pathways. According to the authors, their results suggest that a neutrophil-based assay for analyzing the plasma of suspected septic patients may have clinical relevance.

The study by Chidambaram et al. [5] (GSE58291) analyzed the transcriptomic fingerprint of human corneal tissue with late stage microbial keratitis (the main cause of blindness) caused by fungi and bacteria (including *S. pneumoniae*) against healthy corneal tissue from corpses to identify pathways related with the pathological process. They found a strong correlation between the genes differentially expressed during bacterial and fungal keratitis. Fungal infection unique genes appeared to be related to wound healing response, whereas for bacterial infection they observed an enrichment of HIF1A-induced genes in the bacterial infected corneal tissue.

References

1. van Lieshout, M.H.; Scicluna, B.P.; Florquin, S.; van der Poll, T. Nlrp3 and asc differentially affect the lung transcriptome during pneumococcal pneumonia. *Am J Respir Cell Mol Biol* **2014**, *50*, 699-712.
2. Jonczyk, M.S.; Simon, M.; Kumar, S.; Fernandes, V.E.; Sylvius, N.; Mallon, A.M.; Denny, P.; Andrew, P.W. Genetic factors regulating lung vasculature and immune cell functions associate with resistance to pneumococcal infection. *PLoS One* **2014**, *9*, e89831.
3. Restori, K.H.; Kennett, M.J.; Ross, A.C. Immunization with pneumococcal polysaccharide serotype 3 and lipopolysaccharide modulates lung and liver inflammation during a virulent streptococcus pneumoniae infection in mice. *Clinical and vaccine immunology : CVI* **2013**, *20*, 639-650.
4. Khaenam, P.; Rinchai, D.; Altman, M.C.; Chiche, L.; Buddhisa, S.; Kewcharoenwong, C.; Suwannasaen, D.; Mason, M.; Whalen, E.; Presnell, S.*, et al.* A transcriptomic reporter assay employing neutrophils to measure immunogenic activity of septic patients' plasma. *J Transl Med* **2014**, *12*, 65.
5. Chidambaram, J.D.; Kannambath, S.; Srikanthi, P.; Shah, M.; Lalitha, P.; Elakkiya, S.; Bauer, J.; Prajna, N.V.; Holland, M.J.; Burton, M.J. Persistence of innate immune pathways in late stage human bacterial

and fungal keratitis: Results from a comparative transcriptome analysis. *Front Cell Infect Microbiol* **2017**, *7*, 193.

Supplementary data

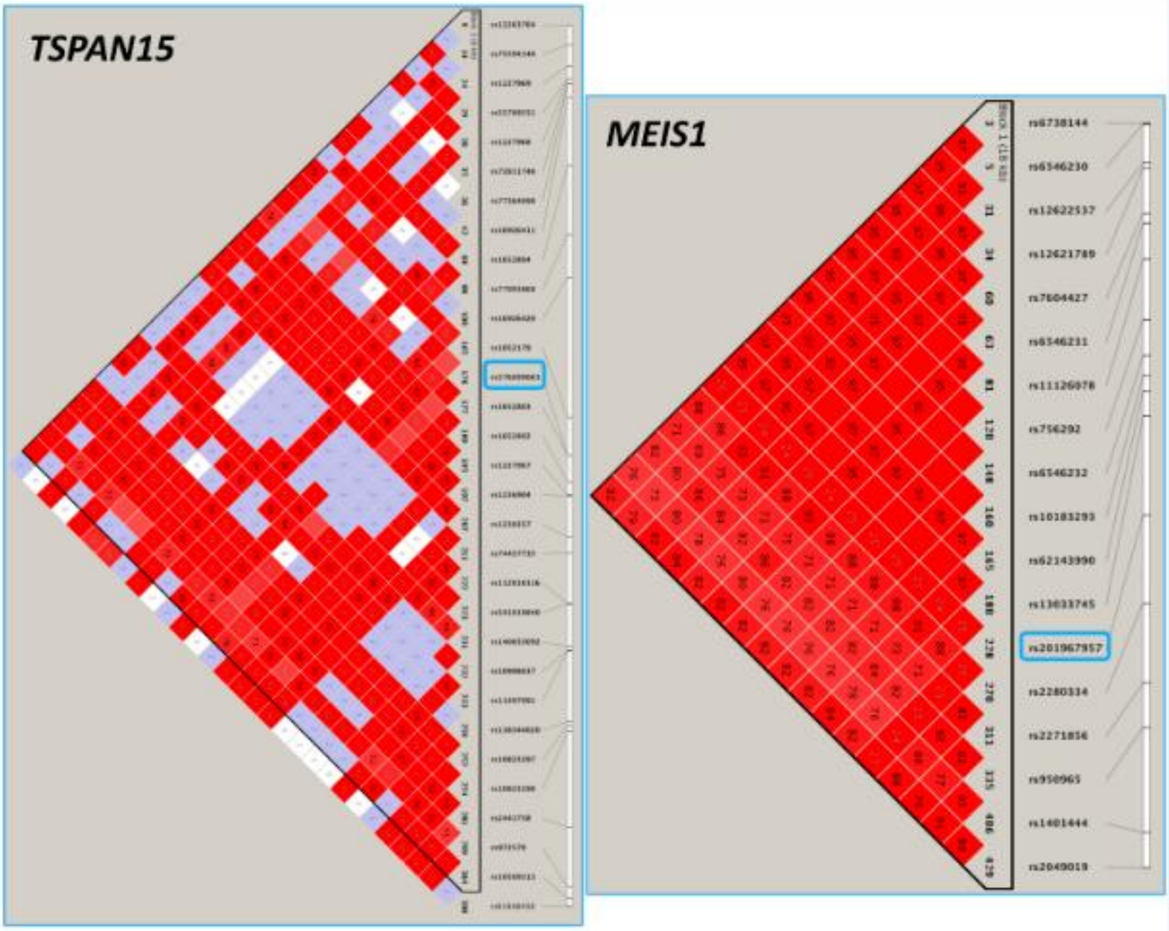

Figure S1. Linkage disequilibrium patterns in gene *MEIS1* and *TSPAN15* for variants with MAF > 5%. The blue arrow on the right of each figure points to the SNP candidates rs201967957 and rs576099063.

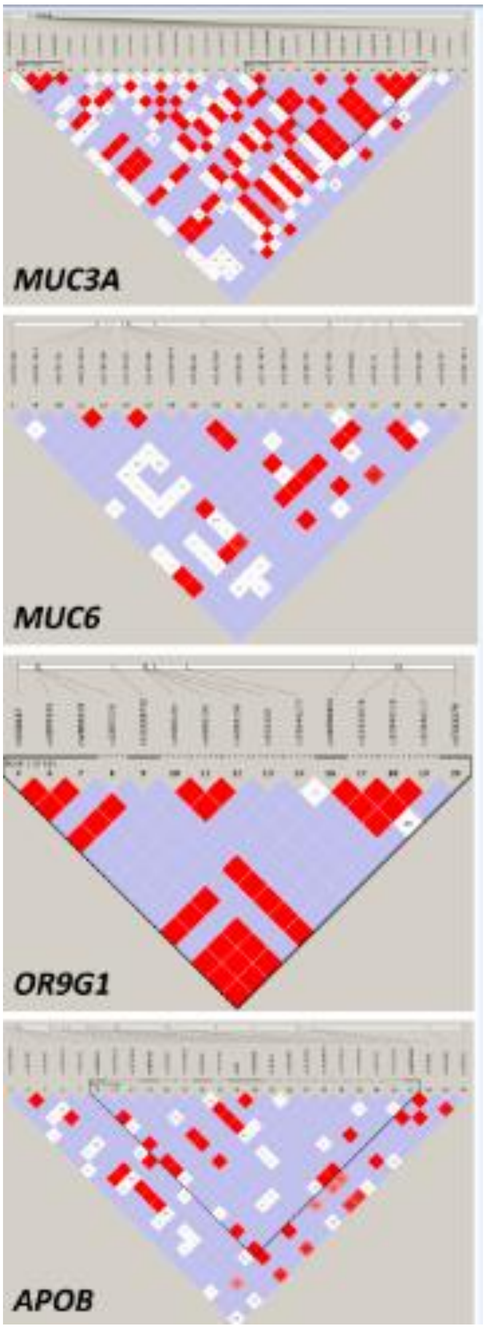

Figure S2. Linkage disequilibrium patterns for all the SNPs observed in genes *MUC3A*, *MUC6*, *OR9G1*, and *APOB*.
